# Supplementary material for: Designing pathways for bioproducing complex chemicals by combining tools for pathway extraction and ranking
Source: Nat Commun. 2025 May 24;16:4839. doi: 10.1038/s41467-025-59827-7 (PMC12103536; doi:10.1038/s41467-025-59827-7)
Supplement: Supplementary file 1 — Supplementary Information [file 41467_2025_59827_MOESM1_ESM.pdf]

Designing pathways for bioproducing complex chemicals by combining tools for pathway extraction and ranking

Sveshnikova and Oftadeh *et al.*

Supplementary Table 1. Case study compounds tested for subnetwork extraction and pathway search. The number of compounds and reactions in the extracted subnetworks for the eight natural case study compounds.

Compound name	SA* score	Number of compounds	Number of reactions
Benzyl benzoate	1.21	8'366	33'579
Benzyl cinnamate	1.54	8'402	33'989
Cinnamoyl serotonin	2.10	8'362	33'656
Berberine	2.79	8'464	34'183
Ajmalicine	3.94	8'390	33'852
Quercetin 3-*O*-(6'-acetyl-glucoside)	4.08	8'434	34'122
Scopolamine**	4.64	8'491	34'410
Strictosidine	4.85	8'389	33'951

* SA: synthetic accessibility.

** After adding the required reactions from the ATLASx and the latest versions of the KEGG and MetaCyc databases.

Supplementary Table 2. Small biological molecules that have already been produced in a host organism have synthetic accessibility (SA) score between 1 (easy to make) and 6.5 (difficult to make).

**Class of compounds**	**Examples (SA score)**
Alkaloids	Berberine (2.79), ajmalicine (3.94), scopolamine (4.64), strictosidine (4.85), staurosporine (4.95)
Products of shikimate pathway	Cinnamoyl serotonin (2.10)
Polyphenols	Rosmarinic acid (2.90), curcumin (2.43), resveratrol (2.11)
Glycosylated isoflavonoids	Quercetin 3-*O*-*N*-acetylglucosamine (4.08)
Oligomeric products of flavonoids	Proanthocyanidins (4.77)
Lactam antibiotics	Ansalactam C (6.5), carbapenem (3.85)
β-lactones	Salinosporamide A (4.82)
Stilbenes	Pterostilbene (1.89)
Fungal indole diterpenes	Aflatrem 4 (6.27)
Phosphonates	Fosfazinomycin (4.3)
Natural colorants	Betanin (5.36), violacein (3.04)

Supplementary Table 3. Comparison of the EC classes match for the pathways predicted with SubNetX with the pathways retrieved from the literature.

Compound	# three-level ECs		# four-level ECs	
	Recovered from literature	Matched	Recovered from literature	Matched
Benzyl benzoate	8	0-4	8	0-2
Benzyl cinnamate	6	2-6	6	0-3
Cinnamoyl serotonin	5	2-4	5	0-4
Berberine	10	5-8	17	3-8
Ajmalicine	12	4-6	14	1-3
Quercetin 3-*O*-(6'-acetyl-glucoside)	9	1-6	10	0-5
Scopolamine	13	6-9	18	4-6
Strictosidine	12	4-6	15	1-2

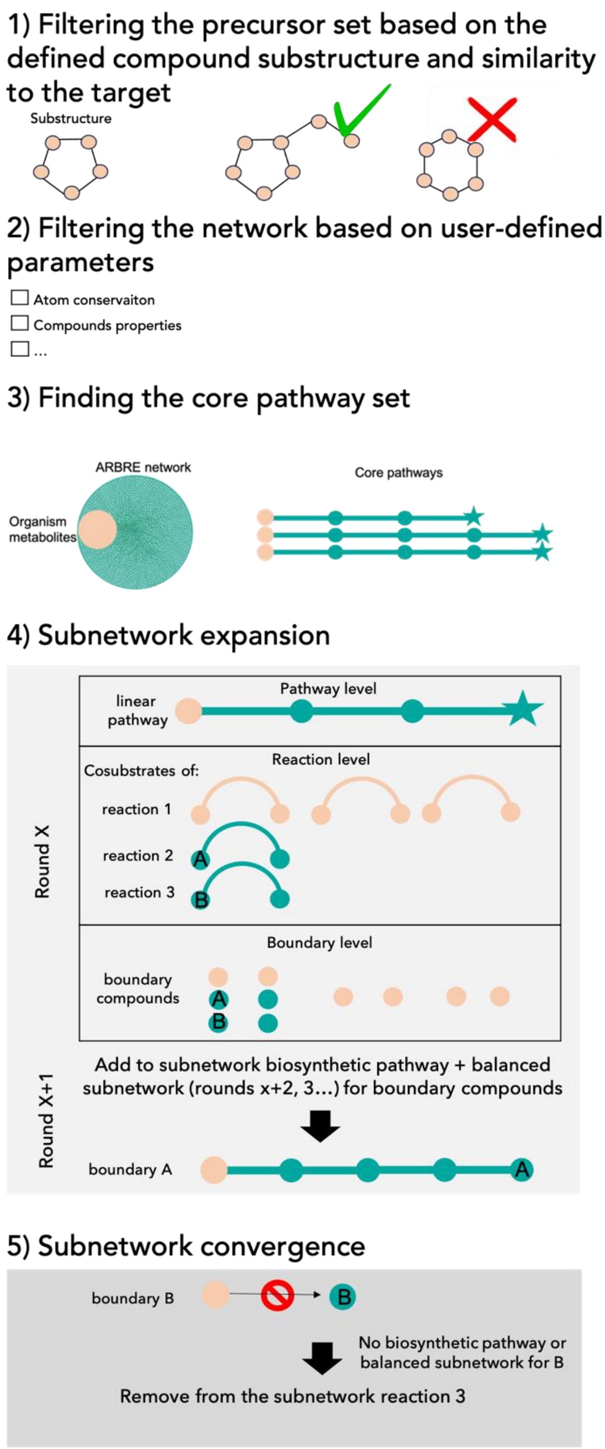

Supplementary Figure 1. Network extraction pipeline. The subnetwork extraction is done in 5 steps (Figure 1): 1) Filtering the precursor set based on the defined compound substructure and similarity to the target; 2) Filtering the network based on user-defined parameters; 3) Finding the core pathway set; 4) Subnetwork expansion; 5) Subnetwork convergence.

a

LCSB ID: 59924115
(3S,5S,6R)-8-methyl-8-azabicyclo[3.2.1]octane-3,6-diol

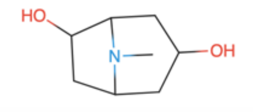

LCSB ID: 3817731
[(7-chloro-3-bicyclo[2.2.1]heptanylidene)amino]urea

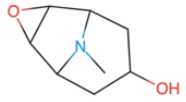

b

Step 1

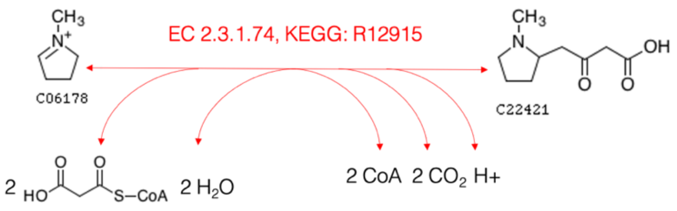

Step 2

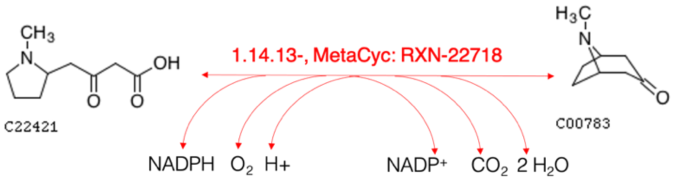

c

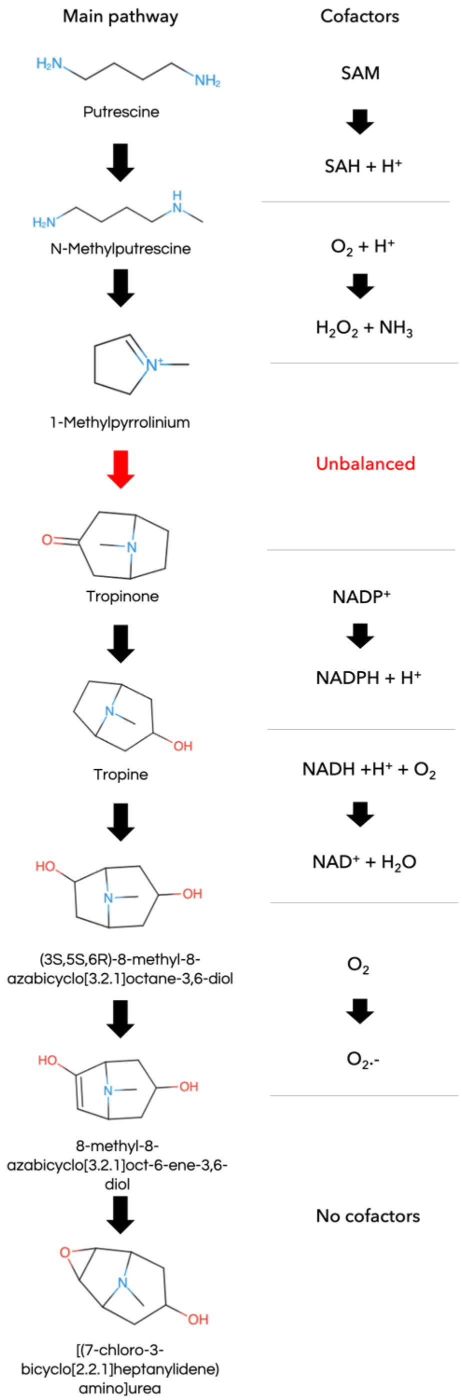

Supplementary Figure 2. Reconstructing putrescine subnetwork a 2 boundary compounds for which additional pathways from putrescine had to be added to ARBRE network to predict balanced subnetwork for scopolamine production from ATLASx network; **b** Two-step reconstruction of the unbalanced reaction in the pathway added from ATLASx; **c** Pathway added from ATLASx.

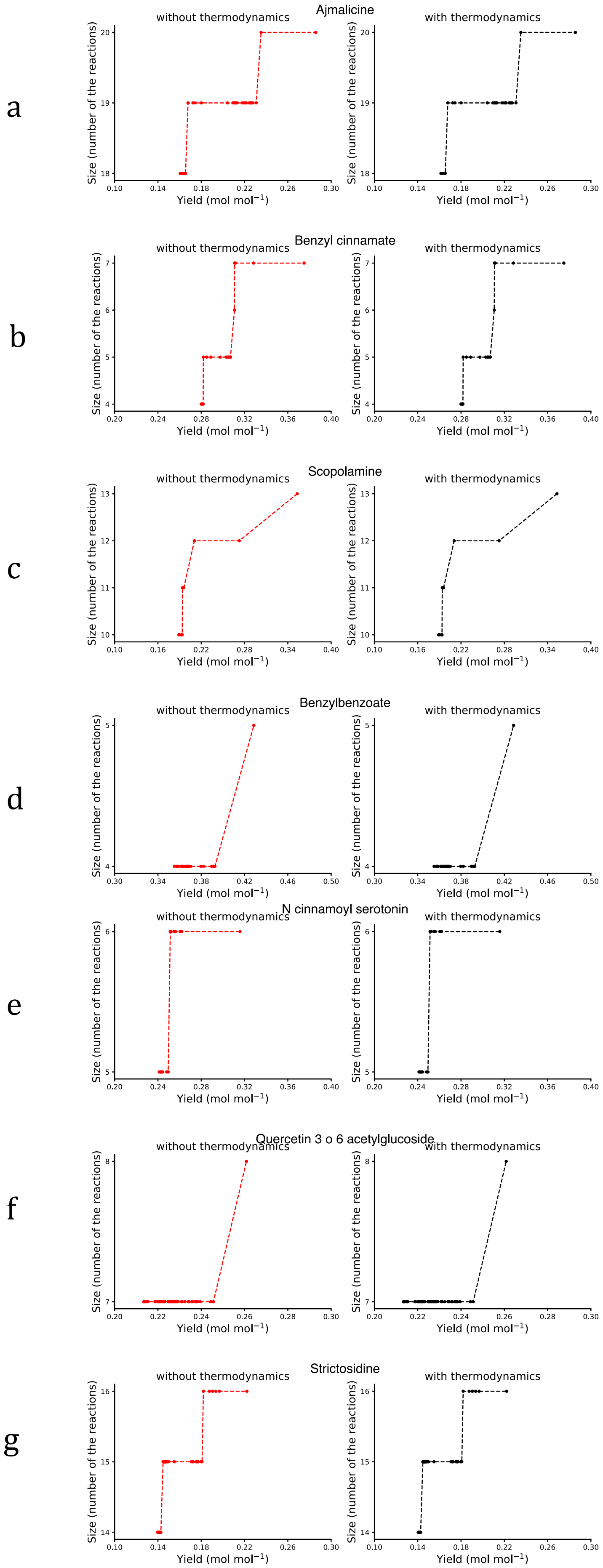
a
Ajmalicine
without thermodynamics
with thermodynamics
Size (number of the reactions)
Yield (mol mol^{-1})
b
Benzyl cinnamate
without thermodynamics
with thermodynamics
Size (number of the reactions)
Yield (mol mol^{-1})
c
Scopolamine
without thermodynamics
with thermodynamics
Size (number of the reactions)
Yield (mol mol^{-1})
d
Benzylbenzoate
without thermodynamics
with thermodynamics
Size (number of the reactions)
Yield (mol mol^{-1})
e
N cinnamoyl serotonin
without thermodynamics
with thermodynamics
Size (number of the reactions)
Yield (mol mol^{-1})
f
Quercetin 3 o 6 acetylglucoside
without thermodynamics
with thermodynamics
Size (number of the reactions)
Yield (mol mol^{-1})
g
Strictosidine
without thermodynamics
with thermodynamics
Size (number of the reactions)
Yield (mol mol^{-1})

Supplementary Figure 3. Exploring the trade-off between size and yield. a ajmalicine **b** benzyl cinnamate, **c** scopolamine, **d** benzylbenzoate, **e** N-cynnamoyl serotonin, **f** quercetin-3-6-acetylglucoside, and **g** strictosidine. Exploring the Pareto fronts generally showed that achieving higher yields requires integrating additional reactions. Integrating thermodynamics impacted the Pareto front only for berberine, while the other compounds showed no difference. Source data are provided as a Source Data file.

a

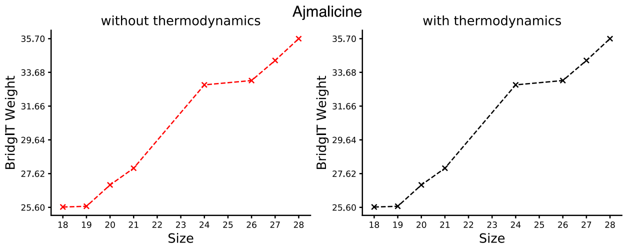
Ajmalicine
without thermodynamics
with thermodynamics
BridgIT Weight
Size
35.70
33.68
31.66
29.64
27.62
25.60
18 19 20 21 22 23 24 25 26 27 28

b

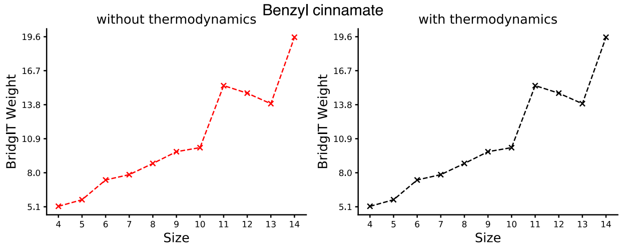
Benzyl cinnamate
without thermodynamics
with thermodynamics
BridgIT Weight
Size
19.6
16.7
13.8
10.9
8.0
5.1
4 5 6 7 8 9 10 11 12 13 14

c

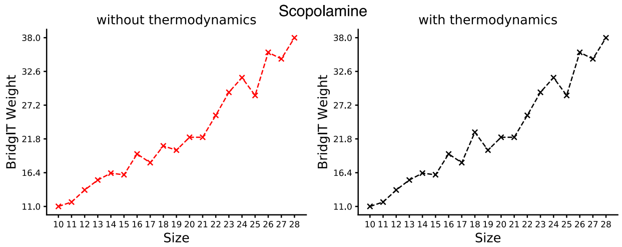
Scopolamine
without thermodynamics
with thermodynamics
BridgIT Weight
Size
38.0
32.6
27.2
21.8
16.4
11.0
10 11 12 13 14 15 16 17 18 19 20 21 22 23 24 25 26 27 28

d

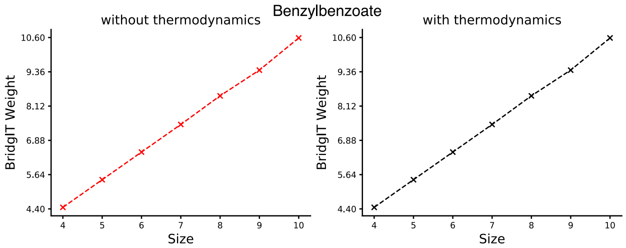
Benzylbenzoate
without thermodynamics
with thermodynamics
BridgIT Weight
Size
10.60
9.36
8.12
6.88
5.64
4.40
4 5 6 7 8 9 10

e

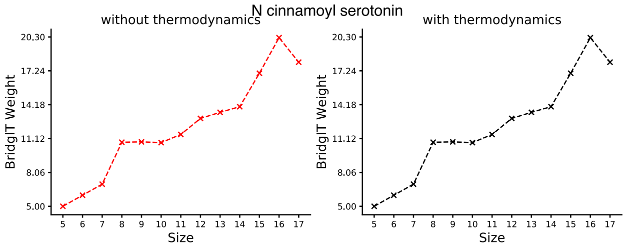
N cinnamoyl serotonin
without thermodynamics
with thermodynamics
BridgIT Weight
Size
20.30
17.24
14.18
11.12
8.06
5.00
5 6 7 8 9 10 11 12 13 14 15 16 17

f

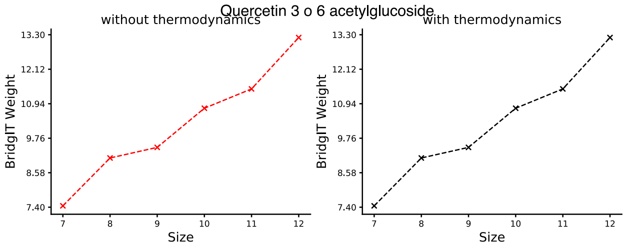
Quercetin 3 o 6 acetylglucoside
without thermodynamics
with thermodynamics
BridgIT Weight
Size
13.30
12.12
10.94
9.76
8.58
7.40
7 8 9 10 11 12

g

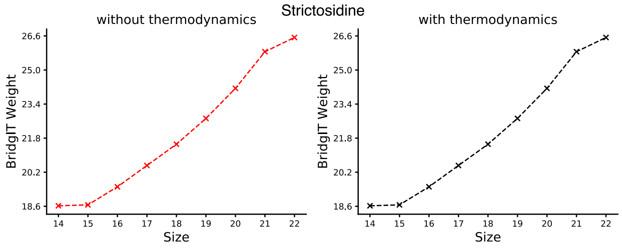
Strictosidine
without thermodynamics
with thermodynamics
BridgIT Weight
Size
26.6
25.0
23.4
21.8
20.2
18.6
14 15 16 17 18 19 20 21 22

Supplementary Figure 4. Exploring the trade-off between size and BridgIT weight. a ajmalicine **b** benzyl cinnamate, **c** scopolamine, **d** benzylbenzoate, **e** N-cynnamoyl serotonin, **f** quercetin-3-6-acetylglucoside, and **g** strictosidine. Although the general trend showed that the minimum BridgIT weight increased with size, such an increase was not monotonic; in some cases, lower BridgIT weights were obtained by increasing the size. Source data are provided as a Source Data file.

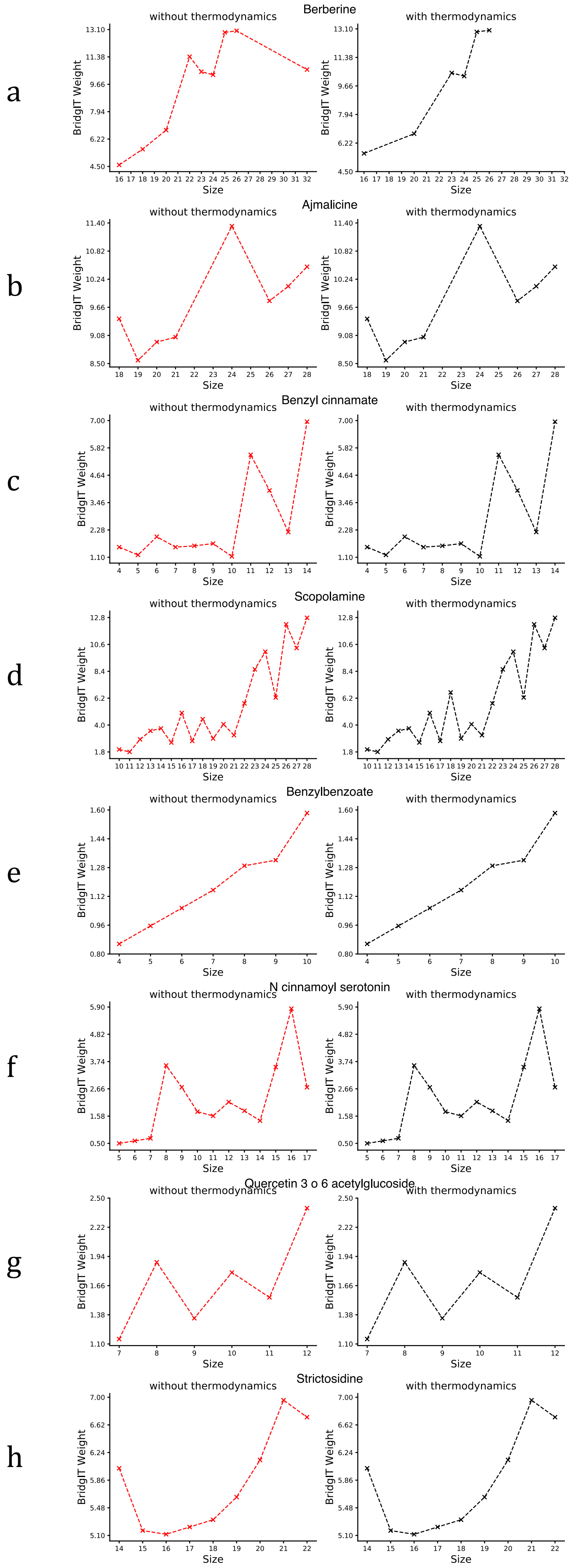
a
Berberine
without thermodynamics
with thermodynamics
BridgIT Weight
Size
b
Ajmalicine
without thermodynamics
with thermodynamics
BridgIT Weight
Size
c
Benzyl cinnamate
without thermodynamics
with thermodynamics
BridgIT Weight
Size
d
Scopolamine
without thermodynamics
with thermodynamics
BridgIT Weight
Size
e
Benzylbenzoate
without thermodynamics
with thermodynamics
BridgIT Weight
Size
f
N cinnamoyl serotonin
without thermodynamics
with thermodynamics
BridgIT Weight
Size
g
Quercetin 3 o 6 acetylglucoside
without thermodynamics
with thermodynamics
BridgIT Weight
Size
h
Strictosidine
without thermodynamics
with thermodynamics
BridgIT Weight
Size

Supplementary Figure 5. Exploring the trade-off between size and BridgIT weight with higher importance assigned to enzyme assignments. **A** berberine, **b** ajmalicine, **c** benzyl cinnamate, **d** scopolamine, **e** benzylbenzoate, **f** N-cynnamoyl serotonin, **g** quercetin-3-6-acetylglucoside, and **h** strictosidine. BridgIT weight is defined as a function of size and BridgIT score, where the latter indicates the quality of enzyme assignments. Here, we assigned a higher weight to BridgIT scores to prioritize finding relevant enzymes. With the new settings, larger pathways had lower BridgIT weights due to prioritizing better enzyme assignments. Source data are provided as a Source Data file.

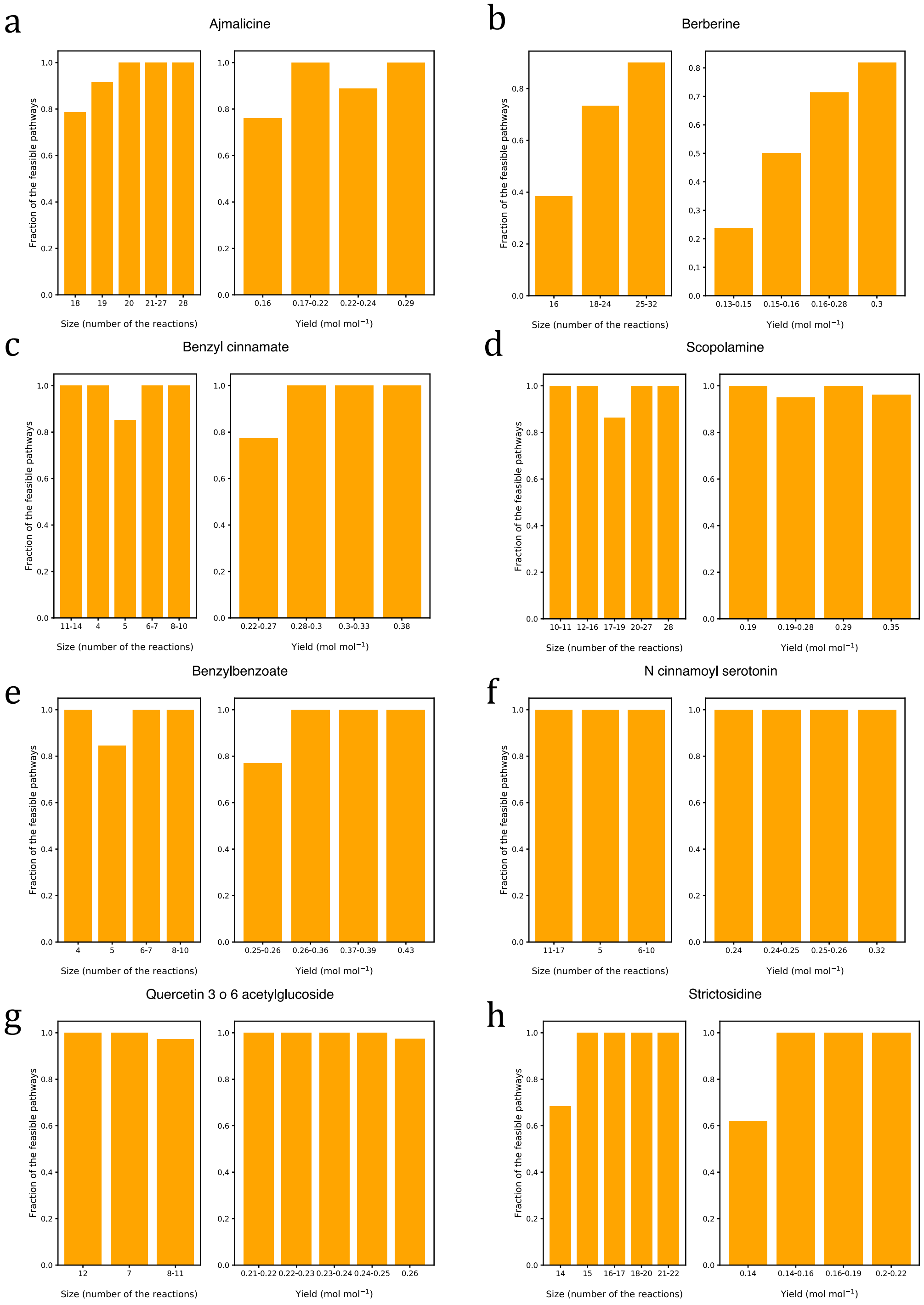

Supplementary Figure 6. Fraction of thermodynamically feasible pathways for different sizes and yields. a ajmalicine, **b** berberine, **c** benzyl cinnamate, **d** scopolamine, **e** benzylbenzoate, **f** N-cynnamoyl serotonin, **g** quercetin-3-6-acetylglucoside, and **h** strictosidine. We did not observe a global trend between thermodynamic feasibility and size or yield. However, compound-specific patterns were observed; thermodynamic feasibility increased with size for ajmalicine and berberine and with yield for benzyl cinnamate and berberine. Source data are provided as a Source Data file.
