# Supplementary material for: Designing pathways for bioproducing complex chemicals by combining tools for pathway extraction and ranking
Source: Nat Commun. 2025 May 24;16:4839. doi: 10.1038/s41467-025-59827-7 (PMC12103536; doi:10.1038/s41467-025-59827-7)
Supplement: Supplementary file 3 — Description of Additional Supplementary Files [file 41467_2025_59827_MOESM3_ESM.pdf]

File Name: Supplementary Data 1

Description: The list of target compounds, their chemical formula, and the size of the minimum pathway for the production of each compound with maximum yield.

File Name: Supplementary Data 2

Description: Comparison between predicted and experimentally implemented pathways for ajmalicine

File Name: Supplementary Data 3

Description: Comparison between predicted and experimentally implemented pathways for scopolamine

File Name: Supplementary Data 4

Description: Comparison between predicted and experimentally implemented pathways for berberine

File Name: Supplementary Data 5

Description: Comparison between predicted and experimentally implemented pathways for benzyl cinnamate

File Name: Supplementary Data 6

Description: Comparison between predicted and experimentally implemented pathways for strictosidine

File Name: Supplementary Data 7

Description: Comparison between predicted and experimentally implemented pathways for quercetin 3-O-(6’-acetyl-glucoside)

File Name: Supplementary Data 8

Description: Comparison between predicted and experimentally implemented pathways for N-cinnamoyl serotonin

File Name: Supplementary Data 9

Description: Comparison between predicted and experimentally implemented pathways for benzyl benzoate
