# Supplementary material for: Designing pathways for bioproducing complex chemicals by combining tools for pathway extraction and ranking
Source: Nat Commun. 2025 May 24;16:4839. doi: 10.1038/s41467-025-59827-7 (PMC12103536; doi:10.1038/s41467-025-59827-7)
Supplement: Supplementary file 13 — Reporting Summary [file 41467_2025_59827_MOESM13_ESM.pdf]

Corresponding author(s): Vassily Hatzimanikatis

Last updated by author(s): Apr 14, 2025

Reporting Summary

Nature Portfolio wishes to improve the reproducibility of the work that we publish. This form provides structure for consistency and transparency in reporting. For further information on Nature Portfolio policies, see our Editorial Policies and the Editorial Policy Checklist.

Statistics

For all statistical analyses, confirm that the following items are present in the figure legend, table legend, main text, or Methods section.

n/a	Confirmed	
[ ]	[x]	The exact sample size (*n*) for each experimental group/condition, given as a discrete number and unit of measurement
[x]	[ ]	A statement on whether measurements were taken from distinct samples or whether the same sample was measured repeatedly
[x]	[ ]	The statistical test(s) used AND whether they are one- or two-sided<br>*Only common tests should be described solely by name; describe more complex techniques in the Methods section.*
[x]	[ ]	A description of all covariates tested
[x]	[ ]	A description of any assumptions or corrections, such as tests of normality and adjustment for multiple comparisons
[x]	[ ]	A full description of the statistical parameters including central tendency (e.g. means) or other basic estimates (e.g. regression coefficient) AND variation (e.g. standard deviation) or associated estimates of uncertainty (e.g. confidence intervals)
[x]	[ ]	For null hypothesis testing, the test statistic (e.g. *F*, *t*, *r*) with confidence intervals, effect sizes, degrees of freedom and *P* value noted<br>*Give P values as exact values whenever suitable.*
[x]	[ ]	For Bayesian analysis, information on the choice of priors and Markov chain Monte Carlo settings
[x]	[ ]	For hierarchical and complex designs, identification of the appropriate level for tests and full reporting of outcomes
[x]	[ ]	Estimates of effect sizes (e.g. Cohen's *d*, Pearson's *r*), indicating how they were calculated

Our web collection on statistics for biologists contains articles on many of the points above.

Software and code

Policy information about availability of computer code

Data collection	Custom code has been written in Python 3.7, and the commercial solver Gurobi was used to solve the MILP problems. The code to generate subnetworks and integrate them into the host models and reproduce the results of this paper is freely available at https://github.com/EPFL-LCSB/subnetx. Installation requires Python 3.7 with the following packages: networkx, rdkit, cobra, and pytfa.
Data analysis	Data analysis has been done with Python 3.7 and the scripts to generate the results are available on our GitHub and GitLab repository.

For manuscripts utilizing custom algorithms or software that are central to the research but not yet described in published literature, software must be made available to editors and reviewers. We strongly encourage code deposition in a community repository (e.g. GitHub). See the Nature Portfolio guidelines for submitting code & software for further information.

Data

Policy information about availability of data

All manuscripts must include a data availability statement. This statement should provide the following information, where applicable:

- Accession codes, unique identifiers, or web links for publicly available datasets
- A description of any restrictions on data availability
- For clinical datasets or third party data, please ensure that the statement adheres to our policy

All the required data including the experimental data collected from the literature is deposited in github (https://github.com/EPFL-LCSB/SubNetX). The code relies on ARBRE database (https://lcsb-databases.epfl.ch/pathways/arbre).The database is also provided separately in the SubNetX repository for completeness. The extracted subnetworks and corresponding models are available in Zenodo (https://doi.org/10.5281/zenodo.15119707).

Research involving human participants, their data, or biological material

Policy information about studies with human participants or human data. See also policy information about sex, gender (identity/presentation), and sexual orientation and race, ethnicity and racism.

Reporting on sex and gender	Not applicable
Reporting on race, ethnicity, or other socially relevant groupings	Not applicable
Population characteristics	Not applicable
Recruitment	Not applicable
Ethics oversight	Not applicable

Note that full information on the approval of the study protocol must also be provided in the manuscript.

Field-specific reporting

Please select the one below that is the best fit for your research. If you are not sure, read the appropriate sections before making your selection.

- [x] Life sciences
- [ ] Behavioural & social sciences
- [ ] Ecological, evolutionary & environmental sciences

For a reference copy of the document with all sections, see nature.com/documents/nr-reporting-summary-flat.pdf

Life sciences study design

All studies must disclose on these points even when the disclosure is negative.

Sample size	We selected 70 target compounds based on two criteria. First, they should be present in the ARBRE network. Second, they should be labeled as drug-like compounds based on ChEBI ontology. We randomly chose 70 compounds while preserving the chemical diversity(by generating random numbers from a uniform distribution). We also subselected eight compounds which their experimental biosynthesis was available in the literature for further analyses. We subselected these compounds while considering the synthetic accessibility to maximize the diversity withing the selected group.
Data exclusions	No data was excluded from the study.
Replication	The code and data are provided to ensure reproducibility.
Randomization	There is no step in the formulation where randomization can be used.
Blinding	There is no step in the formulation where blinding can be used.

Reporting for specific materials, systems and methods

We require information from authors about some types of materials, experimental systems and methods used in many studies. Here, indicate whether each material, system or method listed is relevant to your study. If you are not sure if a list item applies to your research, read the appropriate section before selecting a response.

Materials & experimental systems

n/a	Involved in the study
☒	☐ Antibodies
☒	☐ Eukaryotic cell lines
☒	☐ Palaeontology and archaeology
☒	☐ Animals and other organisms
☒	☐ Clinical data
☒	☐ Dual use research of concern
☒	☐ Plants

Methods

n/a	Involved in the study
☒	☐ ChIP-seq
☒	☐ Flow cytometry
☒	☐ MRI-based neuroimaging

Plants

Seed stocks	This study did not involve plant experiments.
Novel plant genotypes	This study did not involve plant experiments.
Authentication	This study did not involve plant experiments.
